# Association between metabolic syndrome, insulin resistance, and IGF-1 in breast cancer survivors of DIANA-5 study

**DOI:** 10.1007/s00432-023-04755-6

**Published:** 2023-04-27

**Authors:** Mauro De Santi, Giosuè Annibalini, Giuseppe Marano, Giacomo Biganzoli, Elisabetta Venturelli, Massimo Pellegrini, Francesco Lucertini, Giorgio Brandi, Elia Biganzoli, Elena Barbieri, Anna Villarini

**Affiliations:** 1 Unit of Hygiene, Department of Biomolecular Sciences, University of Urbino Carlo Bo, Urbino, Italy; 2 Department of Biomolecular Sciences - Division of Exercise and Health Sciences, University of Urbino Carlo Bo, Urbino, Italy; 3 Department of Clinical Sciences and Community Health and DSRC, University of Milan, Milan, Italy; 4 Epidemiology and Prevention Unit, Department of Research, Fondazione IRCCS Istituto Nazionale dei Tumori di Milano, Milan, Italy; 5 Department of Biomedical, Metabolic and Neural Sciences, University of Modena and Reggio Emilia, Modena, Italy; 6 Hygiene and Public Health, Department of Medicine and Surgery, University of Perugia, Perugia, Italy

**Keywords:** IGF-1, Metabolic syndrome, Breast cancer survivors, Tertiary cancer prevention

## Abstract

**Purpose:**

Circulating insulin-like growth factor-1 (IGF-1) is positively associated with the risk of BC recurrence, and is more frequently dysregulated in older people, especially in those with metabolic syndrome (MetS) and obesity. This study aimed to analyze the association between IGF-1 levels and indices of MetS and insulin resistance in BC survivors.

**Methods:**

Baseline data of 563 BC survivors enrolled in the DIet and ANdrogen-5 (DIANA-5; NCT05019989) study were analyzed.

**Results:**

Lower circulating IGF-1 levels in subjects with MetS than in those without MetS were found. After stratification of the patients according to the diagnosis of MetS, we highlighted that the insulin was the main predictor of elevated IGF-1 levels only in subjects without MetS. Moreover, we found an interaction between high-density lipoprotein cholesterol (HDL-C), glycemia, and IGF-1 levels, showing a positive correlation between HDL-C and IGF-1, especially in subjects with higher values of glycemia and without a diagnosis of MetS.

**Conclusions:**

While IGF-1 levels appear to be much more impaired in subjects diagnosed with MetS, in non-MetS subjects, IGF-1 levels may respond better to metabolic parameters and lifestyle changes. Further studies are needed to analyze the role of physical activity and/or dietary intervention in modulating IGF-1 concentrations in BC survivors.

**Implications for cancer survivors:**

These results could have important clinical implications for planning customized strategies aimed at modulating IGF-1 levels in BC survivors. In fact, while the IGF-1 system seems to be much more compromised in subjects with a diagnosis of MetS, in noMetS subjects, IGF-1 levels could better respond to lifestyle changes.

## Introduction

Epidemiological evidence shows that metabolic syndrome (MetS), fasting hyperinsulinemia, and high levels of circulating insulin-like growth factor-1 (IGF-1) are risk factors for the development and recurrence of breast cancer (BC) (Andò et al. 2019; Calori et al. 2011; Duggan et al. 2011; Endogenous Hormones and Breast Cancer Collaborative Group et al. 2010; Goodwin et al. 2002; Pasanisi et al. 2008).

Insulin and IGF-1 signaling systems are involved in energy metabolism and growth. There is substantial experimental and clinical evidence that cancer cells express insulin and IGF-1 receptors, which are important activators of the Akt and mitogen-activated protein kinase signaling networks in neoplastic tissues (Pollak 2008; De Santi et al. 2016).

MetS is characterized by the presence of at least three out of five dysmetabolic traits according to the consensus definition (incorporating the IDF and AHA/NHLBI definitions) (i.e., abdominal obesity, high blood pressure, low plasma high-density lipoprotein cholesterol (HDL-C), high plasma fasting glucose, and high triglycerides) (Alberti et al. 2009). In epidemiological studies, MetS and its individual components have been associated with BC risk, recurrence, and distant metastasis, after adjusting for stage and hormonal receptor expression (Agnoli et al. 2010; Berrino et al. 2014; Biganzoli et al. 2017; Esposito et al. 2012; Hwang et al. 2020).

Although the exact role of MetS components is largely uncertain, insulin resistance is considered a common mechanism underlying metabolic derangements associated with the syndrome (Cornier et al. 2008; Nelson and Bremer 2010), and it is the most important link between MetS and cancer according to low-grade inflammatory processes (Capasso et al. 2013; Devericks et al. 2022; Duggan et al. 2011; Goodwin et al. 2002). Chronically elevated insulin levels are associated with cell proliferation and survival and contribute to the migration, invasiveness, and metastasis of cancerous cells (Cevenini et al. 2018; Pollak 2008).

The IGF-1 system may also be involved in the effects of hyperinsulinemia on carcinogenic pathways. Indeed, owing to its structural homology to IGF-1, insulin may bind and activate IGF-1 receptor signaling (Doyle et al. 2012; Hankinson et al. 1998; Kaaks 2001). Furthermore, insulin may control IGF-1 production by altering the levels of IGF-1 binding proteins (Lukanova et al. 2001) and/or by hyperinsulinemia-induced promotion of hepatic growth hormone receptor expression and IGF-1 synthesis (Brauna et al. 2011). Accordingly, activated insulin and IGF-1 receptors were detected in all BC subtypes and linked to poor survival in patients with BC (Law et al. 2008).

In the last decade, several studies have revealed an inverse association between circulating IGF-1 levels and MetS in elderly subjects (Brugts et al. 2010) and in large population studies (Oh et al. 2012; Parekh et al. 2010). Moreover, it has been observed that the concentration of IGF-1 decreases with increasing MetS factors (Oh et al. 2012). However, the association between IGF-1 and MetS criteria in BC survivors is not well-known and raises the apparently contradictory finding of a higher risk of BC recurrence in subjects with MetS. Investigation of metabolic and clinical variables in relation to IGF-1 may improve our understanding of this complex onco-metabolic scenario.

Given the role of IGF-1 as a risk factor for BC recurrences, we aimed this study to investigate the association between insulin resistance and MetS risk factors with IGF-1 circulating levels in women with and without MetS (noMetS) from a cohort of BC survivors enrolled in the DIet and ANdrogen-5 (DIANA-5) study (Villarini et al. 2012).

## Materials and methods

### Subjects and methods

The present report concerns the baseline data of 563 women, aged 35–70 years, with early stage invasive BC within the previous 5 years (1.76 years on average), progression-free survivors, recurrence-free, enrolled in the DIANA-5 study, an ongoing multi-center randomized controlled trial aimed at testing the hypothesis that a lifestyle change based on the Mediterranean diet and macrobiotic principles, together with daily moderate intensity physical activity, can reduce the incidence of BC recurrences (ClinicalTrials.gov Identifier: NCT05019989). In the DIANA-5 study were enrolled women at high metabolic and hormonal risk of BC recurrences: ER-negative tumors or serum testosterone level ≥ 0.4 ng/mL (1.338 nmol/mL), and/or serum insulin ≥ 7 μU/mL (50 pmol/L) and/or MetS diagnosis (3/5 risk factors), without a diabetes diagnosis. At sampling time, all enrolled women were in natural or induced menopausal status. Before enrollment, all participants signed an informed consent form, including authorization, to obtain blood samples to study serum parameters. The trial design has been described in detail previously (Villarini et al. 2012).

This study was approved by the Institutional Review Board and Ethical Committee of the National Cancer Institute of Milan (No. 37/07).

### Laboratory analysis

Plasma glucose, triglyceride, and HDL-C levels were measured by routine laboratory techniques. Insulin levels were measured using an immunoradiometric kit (Immunotech, Prague, Czech Republic), which showed intra- and interassay coefficients of variation of 2.2% and 5.1%, respectively, with a mean insulin value of 10 μU/mL.

Serum IGF-1 was measured using commercial radioimmunoassay kits (Biosource, Nivelles, Belgium). Samples were analyzed blinded; the technicians who analyzed the serum samples were unaware of the patients’ disease status. The analyses were organized into 16 analytical batches each consisting of 38 serum samples and 3 quality controls provided by the kit. The interbatch coefficients of variation were 9.29%, 9.61% and 5.19% for IGF I values of 75.16 ng/mL, 144.41 ng/mL and 422.84 ng/mL, respectively.

### Definition of MetS

MetS was defined on the basis of the Consensus definition (incorporating IDF and AHA/NHLBI definitions) with the presence of at least three out of five components, according to the thresholds proposed by the International Diabetic Federation: systolic blood pressure > 130/85 mmHg, fasting plasma glucose ≥ 100 mg/dL (5.6 mmol/L), fasting plasma triglycerides ≥ 150 mg/dL (1.7 mmol/L), HDL-C < 50 mg/dL (1.03 mmol/L), waist circumference ≥ 80 cm (Alberti et al. 2009). As reported in the DIANA-5 study (Villarini et al. 2012), we considered MetS components as well as the presence of treatments for dyslipidemia, hypertension, and hyperglycemia.

### Statistical analysis

Age, anthropometric values, clinical parameters, and therapies were evaluated in the entire group and separately in women with and without MetS. For each numerical variable, the distribution was checked for unimodal and symmetric shapes using graphical procedures (histograms and normal quantile–quantile plots). Variables with approximately unimodal and symmetrical distributions were summarized using means and standard deviations, otherwise through medians and quartiles (Q_1_, Q_3_). The relationship between IGF-1 levels, MetS diagnosis, and the number of MetS components was evaluated using graphical methods (boxplots).

The analyses described below were performed separately for the 206 and 357 women with and without MetS, respectively. The main aim was to evaluate the association of IGF-1 levels with MetS risk factors: waist circumference (cm), IGF-1 concentration (ng/mL), plasma insulin concentration (μU/mL), HOMA1-IR index (fasting plasma glucose (mg/dL) × fasting insulin concentration)/405) (Matthews et al. 1985), systolic and diastolic blood pressure (mm/hg), HDL-C (mg/dL), triglycerides (mg/dL), and glycemia (mg/dL) fasting concentrations. To such end, linear regression models were used, with IGF-1 concentration as the response variable and the remaining variables as independent variables. The association was assessed by evaluating the estimates of the slopes of the regression lines with 95% confidence intervals and association tests (Wald test).

To investigate the joint effects of the above variables on IGF-1, a multivariable model-building procedure based on Harrell’s guidelines (Harrell 2015) was adopted to pursue model robustness and higher generalizability concerning the set of variables and respective effects included in the model. The procedure is outlined as follows:

1. To prevent overfitting, the maximum number of regression coefficients to be estimated by the model was fixed a priori using the 10 subjects-per-coefficient rule.
2. Putative non-linear effects of the independent variables were assessed in univariate and multivariable analysis;
3. A “maximal” model, including the interaction effects of the independent variables, was fitted. Nonlinear effects were no longer considered, because no evidence was found at point 2.
4. The interaction effects were evaluated in the first step using a global ANOVA-like test against the null hypothesis that they were absent in the maximal model. If the null hypothesis could not be rejected, all interactions were removed from the model. Otherwise, if the null hypothesis was rejected, tests for assessing single interactions were performed to determine their presence in the model. Subsequently, a backward variable selection procedure was applied to further reduce the complexity of the model.

During Steps (3) and (4), residual analysis techniques were adopted to check the homoscedasticity, leverage points, and robustness of the model estimates with respect to outliers. The results were reported in terms of estimated regression coefficients and Wald tests, as previously described for univariate models. The interaction effects were represented graphically (effect plots). For each test, statistical significance was set at *P* < 0.05. The analyses were performed using R software version 4.2.1 (R Core Team (2020)—European Environment Agency n.d.) with the rms package (CRAN 2022—Package rms n.d.) and the Knime Analytic Platform version 3.6.0 (Berthold et al. 2021).

## Results

IGF-1 levels were analyzed in 563 women enrolled in the DIANA-5 study. Age, anthropometric values, clinical parameters, and therapies for the MetS and non-MetS groups are shown in Table 1.

**Table 1:** Characteristics of MetS and noMetS groups of BC survivors

	MetS women (*n* = 206)	noMets women (*n* = 357)	Differences between groups (MetS vs noMets)	
			Est (95% CI)	*p* value
AGE (years): mean, SD	54.7, 7.9	50.7, 8.2	4.0 (2.2, 5.0)	< 0.0001
WC (cm): mean, SD	94.5, 10.3	82.1, 10.6	12.4 (10.6, 14.2)	< 0.0001
SBP (mmHg): mean, SD	134.7, 17.6	121.4, 16.9	13.4 (10.4, 16.3)	< 0.0001
DBP (mmHg): mean, SD	87.6, 11.4	79.3, 10.5	8.3 (6.4, 10.2)	< 0.0001
Glycemia (mg/dL): mean, SD	102.4, 19.0	90.6, 9.6	11.8 (9.0, 14.6)	< 0.0001
HDL-C (mg/dL): mean, SD	50.0, 12.1	65.8, 13.7	− 15.9 (− 18.1, − 13.7)	< 0.0001
Triglycerides (mg/dL): median, Q1–Q3	145.5, 103.2–187.8	80.0, 65.0–111.0	65 (53.6–76.4)	< 0.0001
Insulin (µU/mL): median, Q1–Q3	11.3, 8.0–14.9	8.3, 6.3–10.3	3.0 (1.9–4.0)	< 0.0001
HOMA1-IR: median, Q1–Q3	2.8, 1.9–3.9	1.8, 1.4–2.3	0.9 (0.6–1.1)	< 0.0001

Estimates of the difference between the means (medians), with respective 95% CI, and *p* value from the *t* test (or the Mood’s median test)

WC, waist circumference; SBP, systolic blood pressure; DBP, diastolic blood pressure; HDL-C, high-density lipoprotein cholesterol

The average age at study entry was 52.2 years (± 8.3) years, with significantly younger women in the noMetS group (*p* < 0.0001). Of the 563 patients enrolled in the study, 206 (36.6%) had a MetS diagnosis, 241 (43.8%) had only one or two MetS traits, and 116 (20.6%) had none. As expected, the risk factors for MetS and fasting insulin levels were significantly lower in the noMetS group, while the levels of IGF-1 were higher.

Although the relationship between tamoxifen and IGF-1 is complex and not yet completely described, evidence suggests that tamoxifen suppresses IGF-1 plasma levels in early and advanced BC patients (Mandalà et al. 2001). Notably, the percentage of women taking drugs, such as tamoxifen, which can affect IGF-1 levels, was not significantly different between the two groups (46.6 and 46.2 in MetS and NoMetS, respectively, *p* = 0.995).

**Fig. 1:**
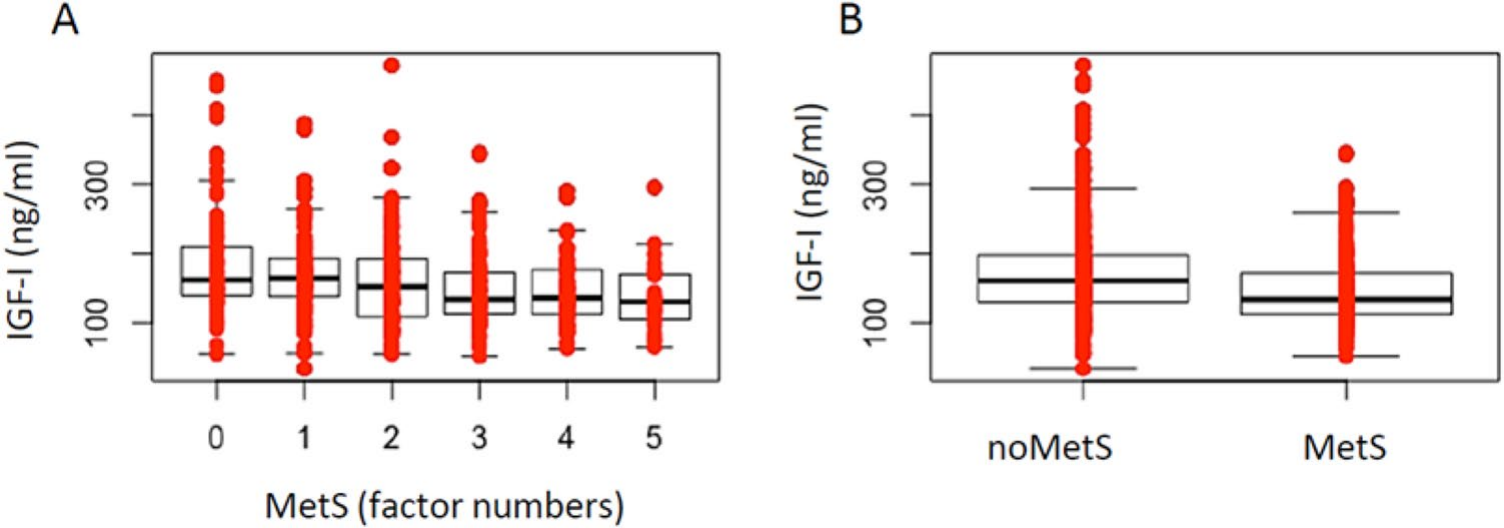
IGF-1 levels in subjects stratifying by MetS factor numbers (**A**) and MetS diagnosis (**B**)

As shown in Fig. 1A, IGF-1 levels decreased with an increase in MetS factor numbers, and were globally lower in women with MetS than in those without MetS (*p* < 0.0001) (Fig. 1B).

The results of univariate analysis are shown in Table 2. In women with and without MetS, there was a significant inverse association between IGF-1 concentration and waist circumference (*p* < 0.0001 and *p* = 0.0098, respectively). The model estimated an average IGF-1 decrease of 1.31 ng/mL (95% CI; − 1.96 to − 0.65 ng/mL) in MetS group and 0.84 ng/mL (95% CI; − 1.46 to − 0.20 ng/mL) in noMetS group for each unit of waist circumference. No significant relationships emerged with the other metabolic characteristics in the MetS group. In the noMetS subjects, a negative association was found for SBP, while a positive association emerged for HDL-C and insulin concentrations. The estimated average IGF-1 decrease was 0.42 ng/mL for a unit increase of SBP (95% CI; − 0.82 to − 0.02 ng/mL; *p* = 0.0377) and an average IGF-1 increase of 0.88 ng/mL for a unit increase of HDL-C (95% CI 0.40–1.36 ng/mL; *p* = 0.0004) and 1.75 ng/mL for a unit increase of insulin (95% CI; 0.32–3.17 ng/mL; *p* = 0.0160). We also observed a positive correlation between HOMA1-IR and IGF-1 levels in noMetS women (Table 2).

**Table 2:** Association of IGF-1 with the independent variables (univariate and multivariable regression analyses)

Univariate models	MetS woman		noMetS woman	
	Est (95% CI)	*p* value	Est (95% CI)	*p* value
WC (cm)	− 1.31 (− 1.96, − 0.65)	< 0.0001*	− 0.84 (− 1.47, − 0.20)	0.0098*
DBP (mm hg)	0.08 (− 0.54, 0.69)	0.8114	− 0.55 (− 1.20, 0.09)	0.0931
SBP (mm hg)	0.04 (− 0.36, 0.43)	0.8630	− 0.42 (− 0.82, − 0.02)	0.0377*
Glycemia (mg/dL)	− 0.05 (− 0.43, 0.32)	0.7724	0.20 (− 0.51, 0.91)	0.5802
HDL-C (mg/dL)	0.29 (− 0.29, 0.86)	0.3279	0.88 (0.40, 1.36)	0.0004*
Triglycerides (mg/dL)	− 0.03 (− 0.11, 0.04)	0.3781	− 0.10 (− 0.23, 0.04)	0.1556
Insulin (μU/mL)	− 0.65 (− 1.72, 0.42)	0.2337	1.75 (0.32, 3.17)	0.0160*
HOMA1-IR	− 2.3 (− 5.89, 1.28)	0.2070	7.83 (1.78, 13.88)	0.0114*

Multivariable model with variable selection	MetS woman		noMetS woman	
	Est (95% CI)	*p* value	Est (95% CI)	*p* value
WC (cm)	− 1.30 (− 1,96, − 0.63)	0.0002*		
SBP (mmHg)			− 0.50 (− 0.89, − 0.11)	0.0133*
Glycemia (mg/dL)	− 2.14 (− 3.90, − 0.37)	0.0185*	− 4.06 (− 7.37, − 0.77)	0.0162*
HDL-C (mg/dL)	− 4.78 (− 8.78, − 0.77)	0.0204*	− 4.78 (− 9.22, − 0.32)	0.0365*
Glycemia (mg/dL)∙HDL-C(mg/dL)	0.05 (− 0.01, 0.09)	0.0132*	0.06 (0.01, 0.11)	0.0114*
Insulin (μU/mL)			1.82 (0.42, 3.22)	0.0111*

WC, waist circumference; SBP, systolic blood pressure; DBP, diastolic blood pressure

*p < 0.05

In the multivariable regression analysis, HOMA1-IR was excluded from the model building procedure because of its extremely high correlation with insulin (*r* = 0.98 for noMetS women; *r* = 0.93 for the MetS women), thus causing multicollinearity-related issues. During the model building phase, the tests for overall interaction effects showed no statistical evidence (*p* = 0.4252 and *p* = 0.0729 for women with and without MetS, respectively). However, in both cases, evidence of an interaction effect between HDL-C concentration and glycemia was found (tests of association under the maximal model: *p* = 0.0012 and *p* = 0.0478 for women with and without MetS, respectively). Therefore, we decided to keep these effects were maintained within the models.

Table 2 presents the results of the final model. The multivariable model revealed that IGF-1 levels were negatively correlated with waist circumference (*p* = 0.0002) and SBP (*p* = 0.0133) in the MetS and noMetS groups, respectively. IGF-1 showed a positive relationship with insulin level (*p* = 0.0111) only in noMetS women. Finally, an interaction between HDL-C and glycemia was found in both groups (MetS, *p* = 0.0132; noMetS, *p* = 0.0114).

**Fig. 2:**
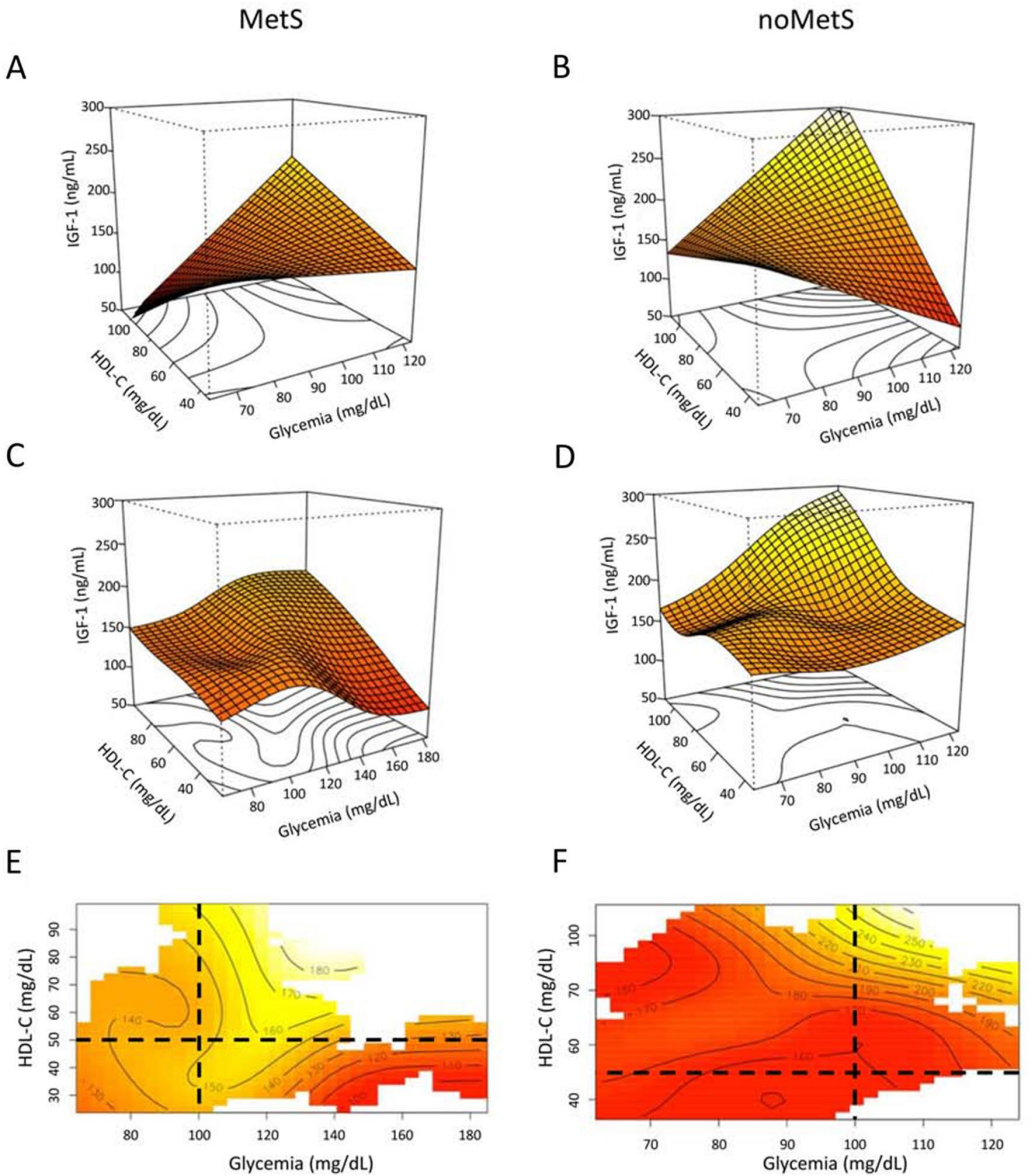
Effect plots showing the joint effect between HDL-C and glycemia on IGF-1 levels according to a linear (**A, B**) and semi-parametric smmothing model (**C–F**). **A, B** Linear interaction effect plots; **C, D**, surface plots; **E, F** contour plots showing the estimates of the average IGF-1 as a function of HDL-C and glycemia. Threshold for glycemia and HDL are shown (dashed lines)

To analyze the interaction effect between HDL-C level and glycemia, the effect plots are shown in Fig. 2. Linear interaction effects, shown in Fig. 2A, B revealed that in subjects with MetS and low HDL-C (e.g., 42 mg/dL), as glycemia increased, a reduction in average IGF-1 levels was observed; however, for higher values of HDL-C (e.g., HDL-C = 56 mg/dL), IGF-1 was positively related to glycemia (Fig. 2A). Similarly, IGF-1 was positively or negatively correlated with HDL-C in subjects with higher (e.g., 107 mg/dL) or lower values of glycemia (e.g., 91 mg/dL) (Fig. 2A). In noMets women, the relationship between average IGF-1 and glycemia was negative for lower HDL-C concentrations (e.g., 55 mg/dL) and positive for higher values (e.g., 75 mg/dL) (Fig. 2B). In Fig. 2B, it may be seen that the relationship between average IGF-1 and HDL-C is positive both for lower (e.g., 85 mg/dL) and higher values (e.g., 96 mg/dL) of glycemia. However, it may be seen that higher slopes of the straight lines within the figure correspond to higher values of glycemia: this suggests that the modification effect of glycemia on the relationship between IGF-1 and HDL-C is analogous to that previously shown for noMets women, although with a lower effect strength.

For a detailed insight into how HDL-C and glycemia are related to IGF-1 levels, a semi-parametric smoothing method (Wood 2017) was used to check for evidence of the linear interaction effect in the parametric model. The surface and contour plots in Fig. 2 show IGF-1 estimates as a function of HDL-C and glycemia. For women with MetS (Fig. 2C, E), the estimated values of IGF-1 were relatively low (approximately within 100–120 ng/mL) when glycemia was within the range of 150–180 mg/dL and HDL-C was lower than 60 mg/dL. When glycemia lies in the range of 100–130 mg/dL, the estimated IGF-1 lies within 140–180 ng/mL and tends to increase with higher values of HDL-C. For lower values of glycemia (lower than 100 mg/dL), the average IGF-1 level again has relatively lower values, approximately 140 ng/mL or lower. For noMetS women, the estimated average IGF-1 showed a different behavior (Fig. 2D, E). In particular, when glycemia is within 80 and 120 mg/dL, average IGF-1 levels increase with HDL-C and can reach levels far higher than those in women with MetS, up to approximately 250 mg/mL (Fig. 2D).

## Discussion

In this study, we explored the relationship between insulin resistance, metabolic indices that characterize MetS, and IGF-1 levels in BC survivors. To this end, we evaluated the relationship between IGF-1 levels and relevant metabolic parameters in women with BC with and without MetS.

Our main findings showed that IGF-1 levels correlated differently with metabolic and insulin resistance-related variables in subjects with or without MetS. Indeed, we found that the insulin level was predictive of elevated circulating IGF-1 levels in women with BC without MetS. We found different levels of IGF-1 associated with glucose and HDL-C levels; in particular, there was a positive association between HDL-C and IGF-1, especially in subjects with higher blood glucose values and without a diagnosis of MetS. In agreement with previous studies, we confirmed lower levels of circulating IGF-1 in participants with MetS and a decrease in IGF-1 levels associated with an increase in MetS criteria (Brugts et al. 2010; Oh et al. 2012; Parekh et al. 2010).

These aspects are particularly interesting in BC survivors, since it is well-known that marked impairments in metabolic function are associated with more aggressive postmenopausal breast tumor biology (Dong et al. 2021; Healy et al. 2010). Jones et al. 2012 (Jones et al. 2012) found that on average, cardiometabolic conditions in BC women are less than those in age-matched sedentary but otherwise healthy women without a history of BC. Remarkably, patients with BC reach a predicted cardiorespiratory condition in a particular age group (e.g., 40 years) approximately 20–30 years earlier than healthy women without a history of BC. It has also been reported that elderly cancer survivors (of mixed diagnoses) have the most functional limitations (are less likely to be able to perform heavy household tasks, walk half a mile, or walk up and down stairs) compared to women without a history of cancer (Elad et al. 2022; Sweeney et al. 2006). In our study, we found a negative correlation between IGF-1 levels and age, BMI, and waist circumferences. The negative correlation between IGF-1 levels and anthropometric measurements is consistent with the results of several epidemiological studies (Gram et al. 2006; Parekh et al. 2010; Roberts et al. 2010; Sandhu et al. 2004; Sesti et al. 2005; Sherlala et al. 2021; Slattery et al. 2005; Succurro et al. 2009). Although the mechanisms involved are not fully understood, it has been proposed that they interact with other metabolic hormones, such as insulin, GH, and leptin (Parekh et al. 2010).

Epidemiological evidence has shown that high circulating IGF-1 levels are positively associated with BC risk (Endogenous Hormones and Breast Cancer Collaborative Group et al. 2010) and prognosis (Murphy et al. 2020; Pasanisi et al. 2008). Moreover, it has been shown that high IGF-1 levels were protective in non-overweight patients but a risk factor for overweight patients (Tong et al. 2020). Notably, as metabolic disorders are frequently associated with low IGF-1 levels in subjects with MetS, it could be hypothesized that IGF-1 is probably not involved as a proliferation inducer but might have other autocrine and/or paracrine actions. In future, we intend to deepen this aspect of the entire cohort of women recruited in the DIANA-5 study.

Interestingly, we observed that IGF-1 levels correlated differently with metabolic and insulin resistance-related variables in subjects with or without MetS. Insulin resistance is often described as a condition of subclinical MetS. Patients can be classified as insulin-resistant, with a HOMA1-IR index ≥ 2.50. An interesting study (Capasso et al. 2013) showed that most BC survivors have normal glucose and insulin levels. However, the measurement of glycemia or insulin alone could underestimate the prevalence of insulin resistance in this population of BC survivors. On the other hand, finding physiological predictors of metabolic changes (e.g., IGF-1), considering glycemia and insulin levels alone, could be more informative than the HOMA1-IR index. This aspect might be relevant for better defining the complicated relationship between MetS, insulin resistance, and IGF-1.

In this study, we observed a positive relationship between HOMA1-IR index and IGF-1 levels in subjects without MetS. In fact, HOMA1-IR was identified as a strong predictor of IGF-1 levels only in the noMetS group. These results are consistent with studies that showed a positive correlation between insulin resistance measures (i.e., HOMA1-IR indices) and levels of circulating IGF-1 (Matsumoto et al. 2018). Furthermore, other studies have highlighted a non-linear (i.e., U-shaped) relationship between insulin resistance and IGF-1 levels in patients with MetS (Friedrich et al. 2012; Oh et al. 2012). It should be noted that there is evidence showing a U-shaped relationship between IGF-1 and general mortality and several chronic degenerative diseases (Zhang et al. 2021). Although this aspect is age-dependent and slightly related to cancer risk, it deserves further investigation.

Interestingly, considering glycemia and insulin levels separately, instead of HOMA1-IR in the regression models, we observed that only insulin levels predicted IGF-1 levels in subjects without MetS, but not in subjects with MetS. In vivo studies suggest that despite the increased uptake of glucose by the tumor, hyperglycemia alone may not increase tumor growth without hyperinsulinemia (Giovannucci et al. 2010; Muti et al. 2002; Vigneri et al. 2009). This suggests that, although many cancer cells rely on glucose metabolism, glucose is not the only key driver of cancer growth and progression in metabolic disorders (Kang et al. 2018), in which other factors such as IGF-1 may be involved.

In this study, we also demonstrated a possible interaction between glycemia and HDL-C levels in relation to circulating IGF-1 levels. Although some studies have failed to find associations between lipoproteins and BC, some large clinical studies have demonstrated a direct association between LDL cholesterol levels and BC risk, and an inverse association between HDL-C and BC risk (Cedó et al. 2019; Strohmaier et al. 2013) and that low HDL-C levels are a single MetS component independently associated with BC recurrence in the DIANA-5 cohort (Berrino et al. 2014). We observed different interaction effects between HDL-C concentration and glycemia in relation to IGF-1 in participants with or without MetS. In women with MetS, we observed that IGF-1 was positively related to HDL-C in those with higher glycemic levels and negatively related to HDL-C in those with lower glycemic levels. In contrast, in subjects without MetS, we observed a strong positive correlation between HDL-C and circulating IGF-1 levels, especially in those with relatively higher values of glycemia. These results highlight the importance of glucose metabolism in the modulation of circulating IGF-1, especially in an oncological context. Intriguingly, an additional complex level of IGF-1 modulation is the presence of a glycosylation site in the Ea peptide of the IGF-1 pro-hormone (Annibalini et al. 2016; Philippou et al. 2014; De Santi et al. 2016). In this context, we recently showed that N-linked glycosylation regulates the stability and secretion of the IGF-1Ea pro-hormone, acting as a glucose-sensitive flexible tail that controls mature IGF-1 production (Annibalini et al. 2018). Moreover, there is mounting evidence for the role of HDL-C in the control of glucose metabolism (Siebel et al. 2015). Although the mechanisms by which insulin resistance contributes to low HDL-C are known, there are also indications that low HDL-C may actually promote the development of diabetes (Drew et al. 2012) and that HDL-C elevation can increase insulin sensitivity in peripheral tissues (Carey et al. 2013). However, there is little evidence of a correlation between IGF-1 levels and circulating lipoproteins. It has been hypothesized a possible role of IGF-1 in regulating HDL-C levels as they show a positive correlation between serum IGF-1 and HDL-C (Song et al. 2016).

Although our results showed an interaction between HDL-C and glycemia, the interpretation is largely incomplete and requires further analysis in a larger population to confirm or exclude the possible role of HDL-C in modulating IGF-1 and its role in BC recurrence. HDL-C is considered as “good cholesterol,” but recent research suggests that this might not always be the case. HDL-C particle composition and biological functions may change under particular conditions, such as metabolic disorders and inflammatory diseases, and a fundamental reassessment of the clinical significance of HDL-C is warranted (März et al. 2017).

An intrinsic limitation of this study is that it does not allow us to clarify cause and effect. However, from a perspective point of view, we hypothesized that lifestyle strategies should focus on the regulation of IGF-1 levels in the normal range rather than on reducing them. The DIANA-5 study will allow us to more thoroughly analyze which factors act as circulating IGF-1 modulators. In the future, we will examine in all women recruited in the Diana-5 study how IGF-1 levels, insulin resistance, and fasting insulin levels in women without MetS may influence the incidence of relapses, secondary BC, and metastases compared to women diagnosed with MetS.

In conclusion, the main findings of this study showed that overall, in women with MetS, mean IGF-1 values are lower and less modulated by metabolic factors than in women without MetS. In particular, findings showing a positive correlation between IGF-1 levels and insulin resistance only in subjects without MetS, a predictive value of circulating IGF-1 for insulin levels, and a possible role of HDL-C in IGF-1 modulation could have important clinical implications for planning customized strategies aimed at modulating IGF-1 levels in BC survivors. In fact, while the IGF-1 system seems to be much more compromised in subjects with a diagnosis of MetS, in noMetS subjects, IGF-1 levels could better respond to lifestyle changes. Further analyses of all DIANA-5 populations are needed to evaluate the role of physical activity and/or dietary intervention in the modulation of IGF-1 concentrations and its implications in BC survivors.

## Data Availability

The raw data supporting the conclusions of this article will be made available by the authors without any undue reservation.
